# HIV-1 Vpr reactivates latent HIV-1 provirus by inducing depletion of class I HDACs on chromatin

**DOI:** 10.1038/srep31924

**Published:** 2016-08-23

**Authors:** Bizhan Romani, Razieh Kamali Jamil, Mojtaba Hamidi-Fard, Pooneh Rahimi, Seyed Bahman Momen, Mohammad Reza Aghasadeghi, Elham Allahbakhshi

**Affiliations:** 1 Cellular and Molecular Research Center (CMRC), Faculty of Medicine, Ahvaz Jundishapur University of Medical Sciences (AJUMS), Ahvaz, 61357-15794, Iran; 2 Department of Biology, Faculty of Science, University of Isfahan, Isfahan, 81746-73441, Iran; 3 Department of Human Viral Vaccines, Razi Vaccine and Serum Research Institute, Karaj, 31976-19751, Iran; 4 Hepatitis and AIDS Department, Pasteur Institute of Iran, Tehran, 13169-43551, Iran; 5 Pilot Biotechnology Department, Pasteur Institute of Iran, Tehran, 13169-43551, Iran

## Abstract

HIV-1 Vpr is an accessory protein that induces proteasomal degradation of multiple proteins. We recently showed that Vpr targets class I HDACs on chromatin for proteasomal degradation. Here we show that Vpr induces degradation of HDAC1 and HDAC3 in HIV-1 latently infected J-Lat cells. Degradation of HDAC1 and HDAC3 was also observed on the HIV-1 LTR and as a result, markers of active transcription were recruited to the viral promoter and induced viral activation. Knockdown of HDAC1 and HDAC3 activated the latent HIV-1 provirus and complementation with HDAC3 inhibited Vpr-induced HIV-1 reactivation. Viral reactivation and degradation of HDAC1 and HDAC3 was conserved among Vpr proteins of HV-1 group M. Serum Vpr isolated from patients or the release of virion-incorporated Vpr from viral lysates also activated HIV-1 in latently infected cell lines and PBMCs from HIV-1 infected patients. Our results indicate that Vpr counteracts HIV-1 latency by inducing proteasomal degradation of HDAC1 and 3 leading to reactivation of the viral promoter.

HIV-1 infection establishes latent viral reservoirs early during primary infection that constitute a major challenge to eradication^1^. Highly active antiretroviral therapy (HAART) can efficiently reduce the detectable viral load in plasma but it cannot eradicate the provirus in latently infected resting CD4+ T cells. Although the exact nature of HIV-1 reservoirs is controversial, it is believed that latent reservoirs of HIV-1 have a very long half-life that ensures a mechanism for life-long persistence of the virus^2^,^3^. The ability of HIV-1 to establish latent infections allows the virus to remain undetected despite antiviral immune responses and antiretroviral therapy^4^.

Following HIV-1 viral entry, the viral genomic RNA is reverse transcribed into proviral cDNA that now has to integrate into the host genome. HIV-1 integrase interacts with cellular proteins such as LEDGF/p75 that guide the pre-integration complex to intronic regions of actively transcribed genes. As a result, HIV-1 proviral cDNA has a tendency to mainly integrate in active regions of the host genome^5^,^6^. The integrated provirus is then chromatinized and similarly to cellular genes, post-translational modifications of histones, such as methylation, acetylation, and phosphorylation, play important roles in regulation of HIV-1 transcription^7^,^8^,^9^,^10^.

Histone acetylation is normally associated with euchromatin and actively transcribed genes^11^,^12^. Several studies have demonstrated that hyperacetylation of core histones on the HIV-1 long terminal repeat (LTR) is correlated with active transcription of HIV-1 genome^10^,^13^,^14^, whereas hypoacetylation of those histones is correlated with HIV-1 latency^15^,^16^. It is well documented that inhibition of histone deacetylases (HDACs) using HDAC inhibitors such as sodium butyrate and SAHA reactivates latent HIV-1 provirus^17^,^18^,^19^. In fact, one of the current strategies to purge the viral reservoirs is reactivating the latent viral reservoirs using HDAC inhibitors, or other small molecules that activate the latent provirus, so that the infected cells could be removed by immune system^20^.

Vpr is a virion-incorporated accessory protein that is conserved among HIV-1 subtypes and other related retroviruses^21^. Vpr has been documented to exert multiple functions such as induction of G2/M cell cycle arrest, induction of apoptosis, and enhancement of viral replication in macrophages^22^,^23^,^24^. It is believed that the interaction of Vpr with Vpr-binding protein (VprBP; also called DCAF1) is essential for most of its biological functions. In fact, Vpr induces proteasomal degradation of a number of proteins by direct interaction with VprBP which consequently engages the Cul4-DDB1[VprBP] E3 ubiquitin ligase^25^,^26^,^27^. We have recently reported that HIV-1 Vpr induces proteasomal degradation of class I HDACs in a localized manner which is more focused on the chromatin. This effect of Vpr was found to counteract silent infection of macrophages by maintaining an active LTR during infection^28^. In this study, we examined whether Vpr has an effect on class I HDACs and reactivation of the HIV-1 provirus in latently infected cells. Using HIV-1 latently infected cell lines and unstimulated PBMCs from patients, we found that expression of Vpr in infected cells or treatment with extracellular Vpr reactivate the latent HIV-1 provirus which was related to depletion of class I HDACs, especially HDAC1 and HDAC3.

## Results

### HIV-1 Vpr induces depletion of chromatin associated class I HDACs in latently infected cells

**Figure 1:**
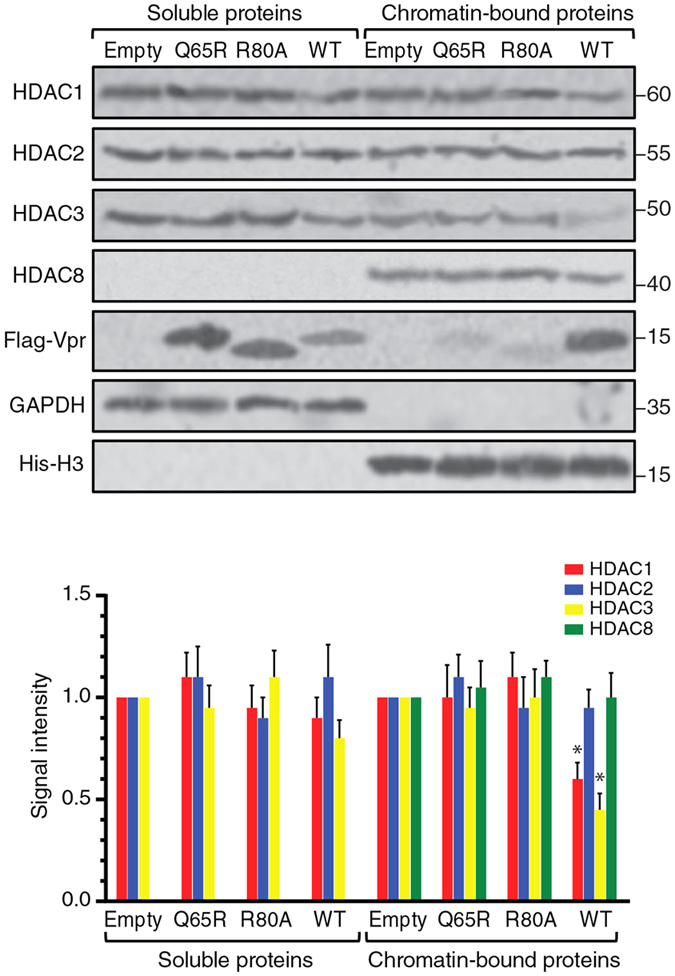
HIV-1 Vpr depletes class I HDACs in J-Lat cells. J-Lat 10.6 cells were transduced with VSV-G pseudotyped mCherry-marked lentiviral vectors for expression of the Flag-tagged wild-type Vpr or the Q65R and R80A mutants. After 48 h, transduced cells were sorted and fractionated into soluble and chromatin-bound proteins.

We have previously reported chromatin depletion of class I HDACs in the presence of HIV-1 Vpr. The effect was found to be proteasome dependent and shown in HeLa cells and primary macrophages. Here, we examined the effect in J-Lat cells as a model for HIV-1 latently infected T cells. J-Lat cells have a copy of GFP-marked latent HIV-1 provirus that could be reactivated upon a variety of stimulations, such as treatment with HDAC inhibitors. A GFP marker is only expressed upon viral activation, helping detection of cells expressing the active virus. We transduced J-Lat cells (clone 10.6) with VSV-G pseudotyped mCherry expressing lentiviral vectors for expression of Flag-tagged Vpr and two Vpr mutants, namely Q65R and R80A that are defective for proteasomal degradation of protein targets of Vpr. As control, cells were also transduced with an mCherry empty vector. Transduced cells positive for mCherry signal were sorted and fractionated into soluble and chromatin-bound proteins (Fig. 1). Western blot analysis of the cellular fractions indicated that HDAC3 had been significantly depleted in the chromatin fraction of J-Lat cells. In fact, expression of the wild type Vpr, but not that of the Q65R and R80A mutants, depleted 55% of HDAC3 in the chromatin fraction. The Q65R and R80A mutants were also less abundant on the chromatin fraction suggesting proper localization of Vpr is required for Vpr-induced proteasomal degradation of HDACs. The wild type Vpr also depleted 40% of HDAC1 in the chromatin fraction. Vpr, however, did not significantly affect HDAC1 and HDAC3 in the soluble fraction. HDAC2 and HDAC8 were not noticeably affected by Vpr in any of the fractions. Of note, this was expected since our previous study also showed that depletion of HDAC2 and 8 only occurs at high expression levels of Vpr, at an MOI of 2.0 or higher^28^. Here we also showed that Vpr was able to induce depletion of all the four members of class I HDACs, including HDAC1, HDAC2, HDAC3, and HDAC8 in J-Lat cells when transduced at an MOI of 5.0 (Supplementary Fig. S1A). But the physiological relevance of this function of Vpr at such high expression levels remains uncertain.

We examined whether Vpr induces depletion of class I HDACs through a VprBP-dependent mechanism. We depleted VprBP in J-Lat cells and transduced them with VSV-G pseudotyped mCherry expressing lentiviral vectors for expression of the wild type Vpr or empty vector. Transduced cells were then sorted and fractionated. As shown in supplementary Fig. S1B, Vpr depleted HDAC1 and HDAC3 only in the presence of VprBP. Depletion of VprBP abrogated the ability of Vpr to induce depletion of HDACs.

To examine whether this effect is reproducible in primary cells, we transduced primary T cells with lentiviral vectors for expression of Vpr and sorted the transduced cells. Analysis of cellular fractions indicated that expression of Vpr downregulated 45% of HDAC3 and 25% HDAC1 in the chromatin fraction (Supplementary Fig. S1C).

### Vpr reactivates HIV-1 in latently infected cells

**Figure 2:**
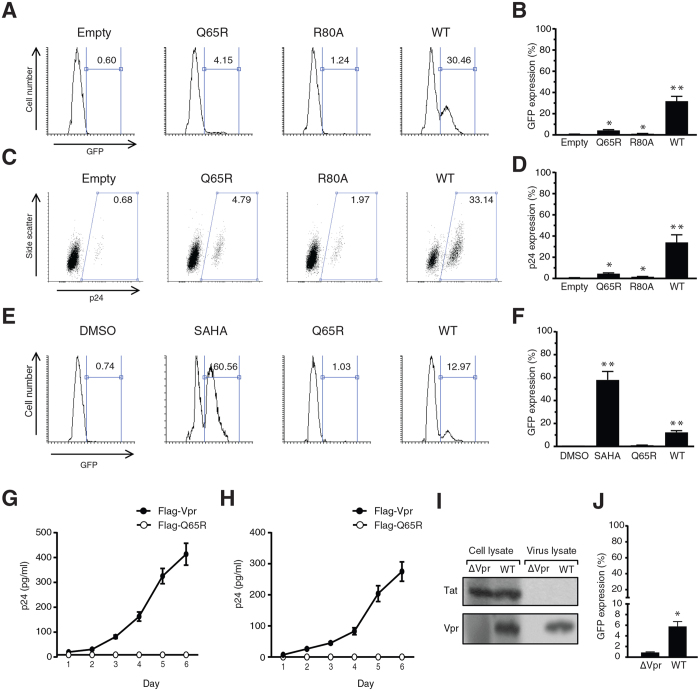
HIV-1 Vpr reactivates latent HIV-1 proviruses. (**A**) J-Lat 10.6 cells were transduced with lentiviral vectors for expression of the wild type or mutant Vpr proteins. After 24 h, cells were analyzed for expression GFP. (**B**) Mean of 3 independent experiments as described in (**A**). (**C**) J-Lat 10.6 cells were transduced with lentiviral vectors for expression of the wild type or mutant Vpr proteins. After 48 h, intracellular p24 was labeled in the transduced cells. (**D**) Mean of 3 independent experiments as described in (**C**). (**E**) J-Lat 10.6 cells were treated with purified Flag-Vpr or Flag-Q65R peptides. Additional cells were also treated with 1 μM SAHA or DMSO as control. After 24 h, expression of GFP was analyzed using flow cytometry. (**F**) Mean of 3 independent experiments as described in (**E**). (**G**) J-Lat 10.6 cells were treated with purified Flag-Vpr or Flag-Q65R peptides for 6 days. Levels of p24 in the supernatant was measured using ELISA. (**H**) U1 cells were treated with Flag-Vpr or Flag-Q65R for 6 days and levels of p24 in the supernatant was measured. (**I**) WT and ∆Vpr HIV-1 were produced in 293T cells and purified from the supernatant using ultracentrifugation. Production and virion incorporation of Vpr and Tat were examined using Western blot in cell lysates and viral lysates. (**J**) J-Lat 10.6 cells were treated with lysates of the WT or ΔVpr HIV-1 virions. All the experiments were repeated 3 times.

It is well documented that inhibition of class I HDACs can reactivate HIV-1 in latently infected cells^17^,^29^,^30^. Since here we found that Vpr induces depletion of HDAC1 and HDAC3 in latently infected cells, we examined whether this effect of Vpr results in reactivation of the latent HIV-1 provirus. We transduced J-Lat cells with lentiviral vectors expressing Flag-Vpr or Q65R and R80A mutants. Reactivation of the latent HIV-1 provirus was measured by analysing GFP expression (Fig. 2A,B). The WT Vpr reactivated the virus in about 30% of the transduced cells. Transduction of J-Lat cells with both Q65R and R80A mutants slightly reactivated the virus, 4.15% and 1.24%, respectively, but the effect of the mutants was significantly lower than that of the WT Vpr. The slight viral reactivation by the mutants could be related to their suboptimal activities translated to viral reactivation while the slight depletion of HDACs by the mutants cannot be detected using the sensitivity of the Western blot analysis.

To further confirm the viral reactivation using a more direct method, cells were immunolabeled for expression of intracellular p24 (Fig. 2C,D). Similarly, the WT Vpr reactivated the virus in about 33% of the transduced cells but the Q65R and R80A mutants reactivated the virus to a much lower extent, 4.79% and 1.97%, respectively. Analysis of the intracellular p24 confirmed the results obtained by analysis of GFP expression in J-Lat cells.

Vpr is incorporated in large amounts into the HIV-1 virions^31^,^32^. Breakdown of HIV-1 virions naturally occurs *in vivo*, resulting in release of Vpr into bodily fluids^33^,^34^. To investigate whether the cell-free Vpr could reactivate the virus in latently infected cells, we treated J-Lat cells with soluble Flag-Vpr or Flag-Q65R peptides and after 24 h, we analysed cells for expression of GFP. As control, we also treated cells with the HDAC inhibitor, SAHA, or DMSO (Fig. 2E,F). The basal rate of virus reactivation in DMSO-treated cells was about 0.74%. Inhibition of HDACs using SAHA reactivated the virus in about 60% of the cells. Vpr reactivated 12.97% of cells. Q65R could only reactivate 1.03% of the cells which was slightly higher than the background but significantly lower than that of the wild-type Vpr. Since both SAHA and Vpr reactivated the virus by affecting HDACs, however through different mechanisms, we then tested whether there could be a synergy between SAHA and Vpr in the context of viral reactivation. As shown in supplementary Fig. S2A, transducing J-Lat cells with lentiviral vectors for expression of Vpr and treatment of J-Lat cells with SAHA reactivated the virus in about 31% and 62% of the cells, respectively. Combination of SAHA and Vpr also reactivated the virus in about 61% of the cells, suggesting the impact of SAHA and Vpr on viral reactivation are not cumulative.

We then examined Vpr-induced viral activation in a time course study and found that treatment of J-Lat cells with Flag-Vpr maintains the virus active throughout a 6 day period of the experiment (Fig. 2G). In addition to J-Lat cells, we tested U1 cells that have been widely used as a model for latently infected monocyte derived cells. Similarly to J-Lat cells, treatment of U1 cells with Flag-Vpr activated the virus and maintained the active expression of the virus for the 6-day period of the experiment (Fig. 2H).

To further examine effect of the virion-incorporated Vpr, which could be released by the virus disruption, we lysed HIV-1 virions. The viral lysates were tested to ensure that they are free of HIV-1 transactivator Tat (Fig. 2I), which could interfere with the experiment. Treatment of J-Lat cells with the disrupted virion suspensions showed that only lysate of the WT virus efficiently reactivated the virus (about 6%), suggesting Vpr released from the WT virions is able to reactivate latently infected cells (Fig. 2J).

In addition to J-Lat clone 10.6 used throughout the study, we transduced J-Lat clone 6.3 with lentiviral vectors for expression of the WT Vpr or its mutants to examine whether the effect of Vpr on viral reactivation is cell-line specific (Supplementary Fig. S2B). We found that expression of Vpr activated HIV-1 in 27.86% of J-Lat 6.3 cells while the virus was reactivated only in a small number of the Q65R and R80A transduced cells. Vpr is also well known for inducing G2 cell cycle arrest^27^,^35^. To examine whether the viral reactivation is resulted from Vpr-induced G2 cell cycle arrest, we transduced J-Lat cells with lentiviral vectors for expression of Vpr. The transduced cells were then treated with caffeine, an ATR inhibitor that inhibits Vpr-induced G2 arrest (Supplementary Fig. S2C). As expected, Vpr induced viral reactivation in about 32% of the transduced J-Lat cells. While caffeine treatment effectively interfered with the ability of Vpr for induction of G2 arrest, it slightly reduced the viral reactivation to 27% (about 15% reduction). This experiment suggested that the ability of Vpr for induction of G2 arrest does not solely account for Vpr-induced viral reactivation but may slightly enhance viral reactivation.

### Vpr-induced reactivation of latent HIV-1 infection is VprBP dependent

**Figure 3:**
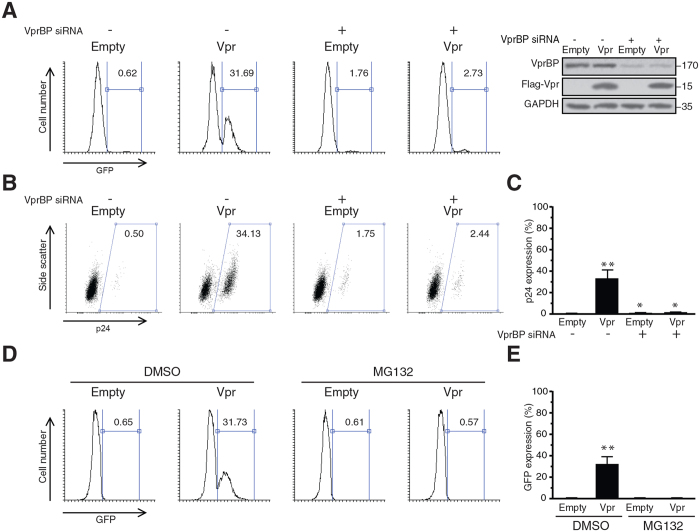
Vpr-induced reactivation of HIV-1 is VprBP and proteasome-dependent. (**A**–**C**) J-Lat 10.6 cells were transfected with siRNA against VprBP or non-targeting siRNA. After 48 h, cells were transduced with lentiviral vectors for expression of Vpr or empty vectors. Twenty four hours post-transduction, cells were analyzed for expression of GFP. Some cells were also spared and analysed using Western blot for depletion of VprBP (**A**). The intracellular p24 was labeled 48 h post-transduction and cells were analysed using flow cytometry (**B**). (**C**) Mean of 3 independent experiments as described in (**B**). (**D**) J-Lat 10.6 cells were transduced with lentiviral vectors for expression of Vpr. Transduced cells were treated with 5 μM MG132 or DMSO. After 24 h, HIV-1 reactivation was analysed by measuring the expression of GFP. (**E**) Mean of 3 independent experiments as described in (**D**).

The finding that the Q65R and R80A mutants were defective for efficient reactivation of the virus suggested that the viral activation by Vpr is VprBP-dependent. But in order to directly examine the VprBP-dependency of this effect, we transfected J-Lat cells with VprBP or non-targeting siRNAs. Transfected cells were then transduced with lentiviral vectors for expression of Flag-Vpr or empty vector. One and two days after transduction, viral reactivation was assessed by analysis of GFP-positive cells (Fig. 3A) and intracellular p24 expression (Fig. 3B,C), respectively. By analysis of GFP-positive cells, we found that expression of Vpr reactivated the virus in 31.69% of the J-Lat cells transfected with non-targeting siRNA. Interestingly, when VprBP was depleted in J-Lat cells, the ability of Vpr to reactivate the virus dropped to 2.72%, suggesting expression of VprBP is required for viral reactivation by Vpr. Depletion of VprBP resulted in viral reactivation in 1.76% of the cells, which was higher than the background control. This increase could be related to the complex functions of VprBP in chromatin regulation or the off-target effects of the VprBP siRNA.

Measuring the intracellular p24 in J-Lat cells also yielded in similar results (Fig. 3B,C). p24 was expressed by 34.13% of the Vpr-transduced cells in the presence of VprBP. But when VprBP was depleted, p24 was expressed by only 2.44% of the Vpr-transduced cells, further confirming the role of VprBP for Vpr-induced reactivation of the latent HIV-1 provirus.

Since VprBP is a component of the E3 ubiquitin ligase complex, dependency of Vpr-mediated viral reactivation on VprBP suggests the proteasome dependency of this effect. To directly demonstrate role of the proteasomal machinery in viral reactivation, we used a proteasomal inhibitor, MG132, to examine viral reactivation by Vpr. We transduced J-Lat cells with lentiviral vectors for expression of Vpr or an empty vector and treated cells with MG132 or DMSO (Fig. 3D,E). As expected, Vpr reactivated the virus in 31.73% of the DMSO-treated cells. However, Vpr was not able to reactivate the virus in MG132-treated cells (0.57%), indicating the proteasome dependency of Vpr-induced reactivation of the virus.

### Depletion of HDAC1 and HDAC3 reactivates latent HIV-1 infections

**Figure 4:**
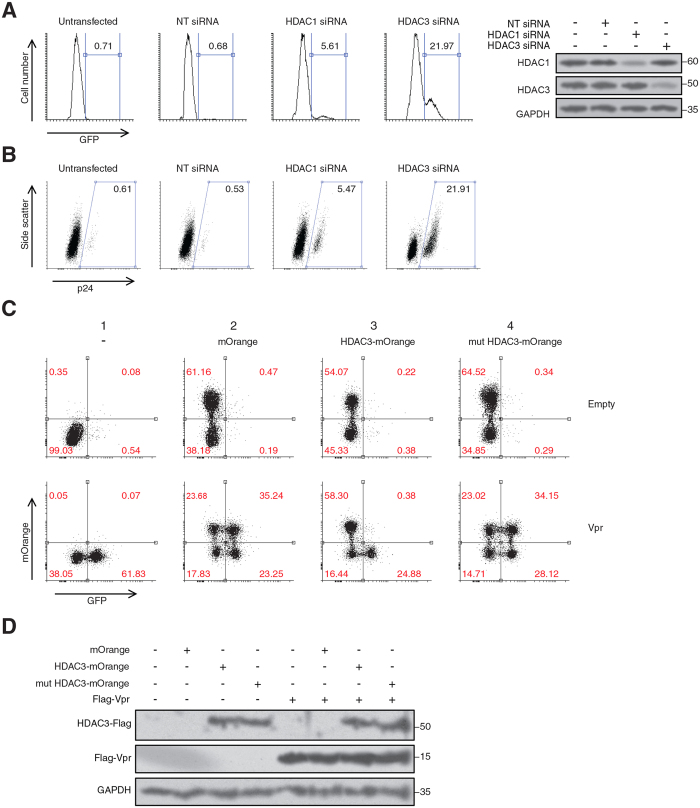
Depletion of HDAC1 and HDAC3 reactivates the latent virus. (**A**,**B**) J-Lat 10.6 cells were transfected with siRNAs against HDAC1 and HDAC3. After 48 h cells were analyzed for expression of GFP (**A**) or intracellular p24 (**B**). The experiments were repeated 2 times and one representative experiment is shown. (**C**) J-Lat 10.6 cells were co-transduced with mCherry-marked lentiviral vectors expressing Vpr and mOrange-marked lentiviral vectors expressing wild-type HDAC3 or H134/135A HDAC3-Flag. After 48 h, cells were analyzed using flow cytometry by gating mCherry expressing cells. HIV-1 reactivation was then assessed using GFP expression among the gated cells. The experiment was repeated 3 times. One representative experiment is shown. (**D**) Western blot analysis of the transduced cells described in (**C**).

We found that expression of HIV-1 Vpr depleted chromatin-associated HDAC1 and HDAC3 in J-Lat cells. We also showed that Vpr reactivated the virus in latently-infected cells. In order to examine whether depletion of HDAC1 and HDAC3 plays a role in viral reactivation, we transfected J-Lat cells (clone 10.6) with siRNAs against HDAC1 and HDAC3 (Fig. 4A,B). By analysis of the GFP expression, we found that siRNA depletion of HDAC1 and HDAC3 in J-Lat cells reactivated the virus in 5.61% and 21.97% of the cells, respectively. Similarly, analysis of intracellular p24 showed that siRNA depletion of HDAC1 and HDAC3 reactivated the virus in 5.47% and 21.91% of the cells, respectively.

To examine whether the observed viral reactivation was dependent on the J-Lat clone, we also depleted HDAC1 and HDAC3 in J-Lat 6.3 (Supplementary Fig. S3). We found that depletion of HDAC1 and HDAC3 reactivated the virus in 8.93% and 30.28% of the transfected cells, respectively. In addition, we also fractionated the transfected cells to investigate where the siRNA depletion of HDACs takes place. Unlike Vpr, siRNA depletion was not specifically directed to chromatin-associated HDACs but affected HDAC1 and HDAC3 on both chromatin and soluble fractions. However, this was the only available technique which could mimic Vpr-induced depletion of HDACs to some extent.

Our results showed a more significant viral reactivation when HDAC3 was depleted. Although this effect could be cell line dependent or rely on efficiency of the depletion, we chose HDAC3 for further analysis since its depletion already showed a higher potency for viral reactivation in our model. We also took advantage of a non-functional HDAC3 that had two point mutations in its deacetylase motif (H134/135A HDAC3-Flag). As shown in supplementary Fig. S4A, mutations were introduced in a conserved motif of HDAC3 based on well described non-functional mutations of HDAC1^36^. Both the wild-type and the mutant HDAC3-Flag were cloned in lentiviral vectors expressing mOrange with the aid of internal ribosomal entry site (IRES). Deacetylase activities of the wild-type and mutant HDAC3 proteins are compared in supplementary Fig. S4B. The histone deacetylase activity of the H134/135A HDAC3 was found completely abolished. We then co-transduced J-Lat cells with lentiviral vectors for expression of Vpr and the wild-type HDAC3 or H134/135A HDAC3. After 48 h, we examined viral reactivation using GFP signal (Fig. 4C,D). In the absence of Vpr (Fig. 4C, upper panels), there was no considerable reactivation of the latent provirus regardless of expression of the empty vector (mOrange), wild-type HDAC3, or the mutant HDAC3 (H134/135A HDAC3). When Vpr was expressed alone (column 1, lower panel), it reactivated the virus in 61.83% of the Vpr-expressing cells. When Vpr was co-transduced with mOrange (Column 2, lower panel), it reactivated the virus in mOrange expressing and non-expressing cells, 35.24% and 23.25%, respectively. Co-transduction of Vpr with the wild-type HDAC3 (column 3, lower panel) resulted in two populations that did or did not express the exogenous HDAC3. Interestingly, in the populations that expressed the exogenous HDAC3 (positive for mOrange), there was no significant reactivation of the virus (0.38%). However, in the population that did not express HDAC3 (negative for mOrange), Vpr reactivated the virus in 24.88% of the cells, suggesting that the complementation of the latently infected cells with exogenous HDAC3 abolishes the ability of Vpr for viral reactivation. Furthermore, co-transduction of Vpr and the non-functional mutant HDAC3 (Column 4, lower panel) also resulted in two populations that did or did not express the mutant protein. But the mutant form of HDAC3 did not affect the virus reactivation by Vpr in either of the populations, suggesting the deacetylase activity of HDAC3 is essential for maintaining HIV-1 latency.

### Vpr binds the HIV-1 LTR in a VprBP-dependent manner

**Figure 5:**
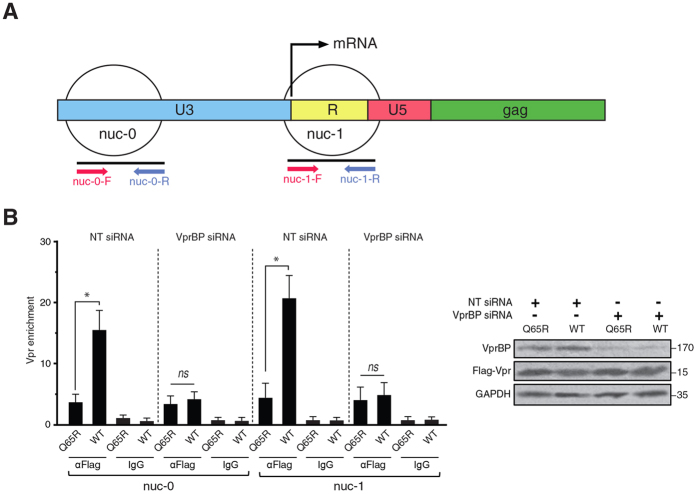
Vpr is recruited to the HIV-1 LTR through VprBP. (**A**) Schematic presentation of nuc-0 and nuc-1 on the HIV-1 LTR and the corresponding primers. (**B**) J-Lat 10.6 cells were transfected with VprBP or non-targeting siRNAs. After 24 h, cells were treated with Flag-Vpr or Flag-Q65R peptides. Twenty four hours after the treatment, chromatin was extracted and sheared by sonication. Chromatin was then pulled down using Flag antibodies or IgG isotype control and after DNA extraction, was subjected to qPCR against nuc-0 and nuc-1. The data are presented as fold enrichment of Vpr on nuc-0 and nuc-1 relative to an intergenic region. The right panel shows Western blot analysis of the cell lysate. The experiment was repeated 3 times.

We showed that Vpr depletes class I HDACs and reactivates latent HIV-1 provirus. Proviral reactivation requires an active promoter. We asked whether there is a level of specificity by Vpr for the HIV-1 LTR. Previous studies have shown two nucleosomes on the HIV-1 LTR, namely nuc-0 and nuc-1, which tightly regulate HIV-1 expression^10^,^37^ (Fig. 5A). In order to examine the interaction between Vpr and the HIV-1 LTR, we focused on binding of Vpr to nuc-0 and nuc-1 by chromatin immunoprecipitation (ChIP) of Vpr. In the same experiment, we also examined involvement of VprBP in this process by transfecting J-Lat cells with VprBP or non-targeting siRNAs. Cells were then treated with Flag-tagged Vpr or Q65R peptides. Chromatin was then extracted and ChIPed using Flag antibody. As shown in Fig. 5B, the WT Vpr was considerably enriched on both nuc-0 and nuc-1 compared to the Q65R mutant. Interestingly, depletion of VprBP abolished enrichment of Vpr on the HIV-1 LTR, indicating Vpr recruitment to the HIV-1 LTR is VprBP dependent. Although a higher enrichment was found for Vpr on nuc-1, a comparison between nuc-0 and nuc-1 was not feasible because the pulled down chromatin in the ChIP assay could contain both nuc-0 and nuc-1 on the same fragments.

### Vpr depletes HDAC1 and HDAC3 on the HIV-1 LTR to activate the HIV-1 promoter

**Figure 6:**
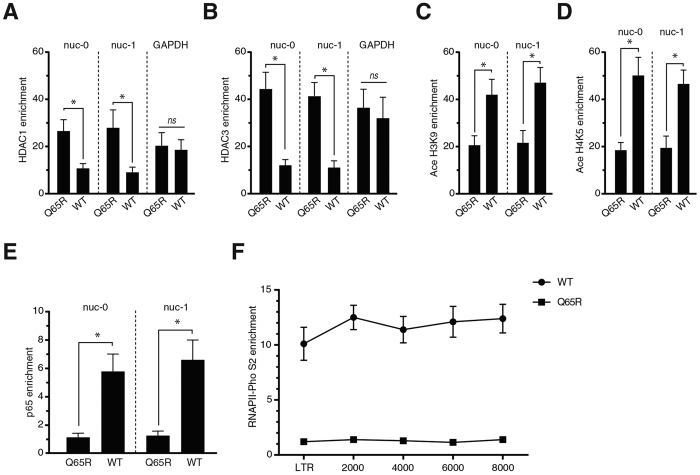
Vpr induces depletion of HDAC1 and HDAC3 on the HIV-1 LTR, resulting in recruitment of markers of active transcription. (**A**–**E**) J-Lat 10.6 cells were treated with Flag-Vpr or Flag-Q65R peptides. After 24 h, the GFP-positive cells were sorted from the Flag-Vpr treated cells. For the Flag-Q65R treated cells, which did not have a significant number of GFP-positive cells, total population was sorted. Cells were lysed and chromatin-immunoprecipitated using antibodies against HDAC1 (**A**) and HDAC3 (**B**) Ace H3K9 (**C**) and Ace H4K5 (**D**) and p65 subunit of NF-κb (**E**). The pulled down DNA was purified and subjected to quantitative PCR using primers against nuc-0 and nuc-1. (**F**) J-Lat 10.6 cells were treated and sorted as described for Fig. 6A–E. Chromatin was immunoprecipitated using antibodies against RNAPII-Pho S2. Purified DNA was then subjected to quantitative PCR using primers against different regions of HIV-1 genome. The data are presented as fold enrichment of the respective proteins relative to their enrichment on an intergenic region. All the graphs show mean of at least 3 independent experiments.

Previous studies have shown that HIV-1 reactivation is associated with decreased occupancy of the HIV-1 LTR by HDAC1 and HDAC3^38^,^39^,^40^. To examine whether Vpr affects binding of HDAC1 and HDAC3 on the HIV-1 LTR of J-Lat cells, we assessed occupancy of HDAC1 and HDAC3 on the nuc-0 and nuc-1 regions (Fig. 5A). J-Lat cells were then treated with purified Flag-Vpr or Flag-Q65R peptides. Of note, peptide treatment was used instead of transduction to avoid introduction of the lentiviral vector LTR into J-Lat cells that could affect the accuracy of the ChIP assays. The GFP positive cells were sorted from the Flag-Vpr treated cells. Since the Flag-Q65R treated cells did not produce a considerable number of GFP positive cells, we used the total population of the Flag-Q65R treated cells for ChIP analysis. Using ChIP, we then analyzed occupancy of HDAC1 and HDAC3 on nuc-0 and nuc-1 (Fig. 6A). Comparison of the WT Vpr with the Q65R mutant indicated that the WT Vpr had depleted HDAC1 about 2.3 and 3 fold, on nuc-0 and nuc-1, respectively. Similarly, Vpr depleted HDAC3 about 3.75 and 3.8 fold on nuc-0 and nuc-1, respectively (Fig. 6B). Interestingly, Vpr did not affect the levels of HDACs on the GAPDH gene (Both Fig. 6A,B), suggesting that Vpr possesses some specificity for targeting HDACs on the HIV-1 promoter.

Modulation of HDAC levels on promoters could affect the acetylation status of nucleosomes. We therefore examined acetylation of histones using antibodies against acetylated histone H3 (Ace H3K9) and acetylated histone H4 (H4K5). As shown in Fig. 6C,D, Vpr induced hyperacetylation of both histone H3 and H4 on nuc-0 and nuc-1 for about 2 to 2.5 fold.

Hyperacetylation of histones on promoters is normally associated with recruitment of transcription factors followed by active transcription of genes. To further study role of Vpr in the HIV-1 LTR activation, we examined recruitment of p65 subunit of NF-κB to the LTR (Fig. 6E). Interestingly, Vpr enriched p65 on nuc-0 about 5.3 fold, suggesting the active transcription of the HIV-1 LTR in the presence of Vpr. To directly examine active transcription of the HIV-1 LTR, we conducted a ChIP assay against the active form of RNA polymerase II, S2 phosphorylated RNAPII (Fig. 6F). ChIP assay indicated 10–12 fold increase in the recruitment of RNAPII to the HIV-1 promoter and coding regions of the viral genome. Taken together, enrichment of p65 and RNAPII on the HIV-1 genome indicated active expression of the viral genome in the presence of Vpr.

### Ability of Vpr for HIV-1 reactivation is conserved among different subtypes of HIV-1

**Figure 7:**
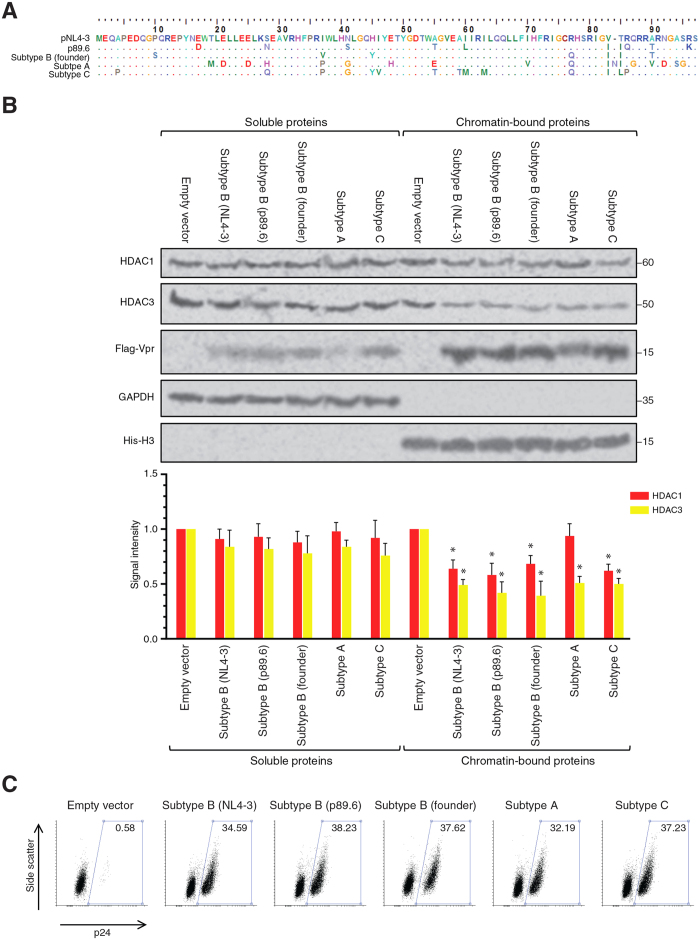
Vpr of other HIV-1 strains also deplete HDACs and reactivate the latent provirus. (**A**) Amino acid alignment of the tested Vpr proteins. Dots represent identity to the pNL4-3 sequence and the only dash, at position 84, represents a gap (**B**) J-Lat 10.6 cells were transduced with mCherry-marked lentiviral vectors for expression of Vpr proteins of different strains and subtypes including the X4 tropic subtype B strain (pNL4-3), dual tropic subtype B strain (p89.36), subtype B founder virus, subtype A, and subtype C. After 48 h, mCherry-expressing cells were sorted and fractionated for Western blot analysis. (**C**) J-Lat 10.6 cells were transduced as described for Fig. 7B. After 48 h, cells were labeled for intracellular p24. Both experiments were repeated 3 times. One representative experiment is shown.

The Vpr sequence used throughout our study was originally cloned from the lab adapted strain pNL4-3, which is an X4-tropic subtype B clone. Sexual transmission of HIV-1, however, is normally established by an R5-tropic founder virus. Other subtypes of HIV-1, such as subtype A and C, have also established themselves as the main cause of HIV pandemic in many regions. In order to explore Vpr-induced viral reactivation in a broader context, Vpr genes of the R5-tropic subtype B p89.6, a subtype B founder strain, a subtype A strain and a subtype C strain were cloned in a lentiviral vector expressing the new Vpr clones as Flag-tagged proteins (See the alignment in Fig. 7A). The new constructs were then used to produce lentiviral vectors for transduction of J-Lat cells. Forty eight hours after transduction, mCherry-positive cells were sorted. As shown in Fig. 7B, the sorted mCherry-positive cells expressed the Flag-tagged Vpr proteins and depleted HDAC3 in chromatin fraction. HDAC1 was also depleted to a lesser extent by all clones of Vpr proteins but subtype A clone, which expressed a lower level of Flag-Vpr.

After showing depletion of HDAC1 and HDAC3 by Vpr proteins of different strains, we tested the ability of different Vpr proteins for viral reactivation. J-Lat cells were transduced with lentiviral vectors expressing Vpr proteins and 48 h later, cells were examined for viral reactivation using immunolabeled intracellular p24 (Fig. 7C). All the 3 strains of subtype B, as well as subtype A and C, efficiently reactivated the latent provirus in about 32–38% of the transduced cells. Slight differences were observed among different clones that could be resulted from transduction efficiency and the expression levels of the different clones. Taken together, the experiment suggested that the ability of Vpr to induce proteasomal degradation of HDAC3 and reactivate the latent HIV-1 is conserved among different subtypes of HIV-1.

### Serum Vpr of HIV-1-infected individuals reactivates latently infected cells

**Figure 8:**
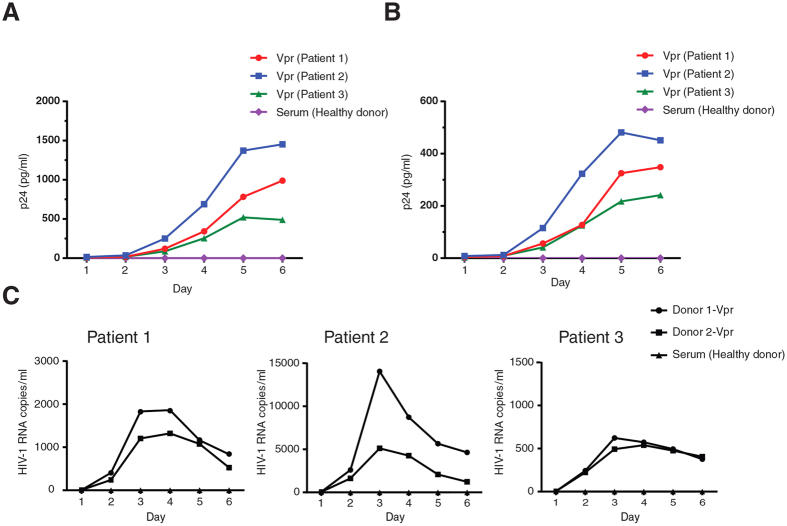
Serum Vpr reactivates HIV-1 latently infected cells. (**A**,**B**) Vpr was purified from the serum of 3 different HIV-1-infected patients (patient 1–3) and used to treat J-Lat 10.6 (**A**) and U1 (**B**) cells. p24 was measured in the supernatant of the treated cells. (**C**) PBMCs were isolated from three HAART-treated HIV-1-infected individuals who had no detectable viral load. Unstimulated PBMCs were then treated with Vpr proteins purified from the serum of two patients, named donor 1-Vpr and donor 2-Vpr. Viral RNA was measured in the supernatant of the treated cells. Serum from a healthy donor was used as control in all the experiments.

Extracellular Vpr is frequently found in the serum of HIV-1 infected individuals. To study the role of serum Vpr in viral reactivation, we needed to purify Vpr from plasma of HIV-1 infected patients. We developed and validated a Vpr antibody that was able to detect Vpr of local HIV-1 isolates (Supplementary Fig. S5A). Using our Vpr antibody, Vpr was then affinity purified from the serum of HIV-1-infected individuals and compared with purified Flag-Vpr (Supplementary Fig. S5B–D). Both serum-purified Vpr and Flag-Vpr were capable of viral reactivation in J-Lat cells. However, serum Vpr had a higher potency for viral reactivation that could be related to the sequence of the clinical isolate or the reduced activity of Flag-Vpr caused by the Flag tag. After confirmation of the Vpr purification procedure, J-Lat cells were treated with the serum Vpr of 3 HIV-1 infected patients (Fig. 8A). Vpr proteins of all the three patients reactivated the latent provirus as measured by the amount of p24 in the supernatant. Vpr was also able to maintain the proviruses active for the 6-day period of the experiment. Similarly, serum Vpr also reactivated the latent provirus in U1 cells (Fig. 8B), indicating the Vpr-induced viral reactivation was not cell-line dependent. Since disruption of HIV-1 virions is likely to be the source of serum Vpr, we used HIV-1 viral lysate to treat unstimulated PBMCs isolated from five HAART-treated HIV-1-infected individuals with no detectable viral loads (Supplementary Fig. S6). Interestingly, lysate of the WT virus activated the virus in the PBMCs of all the patients, as measured by the amounts of viral RNA in supernatant.

In order to examine the effect of Vpr in a more physiological context, Vpr was affinity purified from the serum of two patients. Unstimulated PBMCs of three HAART-treated HIV-1-infected individuals with no detectable viral loads were then treated with the affinity purified Vpr proteins (Fig. 8C). Vpr from both patients reactivated the virus in PBMCs of all the patients but to different extents that could be related to variable amounts of Vpr protein found in the serum of different patients.

## Discussion

The inhibitory effect of HDACs on viral replication has been indicated for a number of DNA viruses^41^,^42^,^43^,^44^, as well as retroviruses^30^,^45^. In addition, some of those viruses have been demonstrated to have evolved mechanisms to counteract the inhibitory effects of HDACs. For instance, the adenovirus Gam1 protein inhibits histone deacetylation by HDAC1^41^. Furthermore, HDACs have been shown to play important roles in controlling latency in some herpesviruses^46^. Herpesvirus ICP0 reportedly inhibits HDAC activity by interacting with the HDAC1 binding partner CoREST^47^. In the case of HIV-1, the importance of HDACs in viral latency is well described^30^,^48^ but proteasome dependent counteraction of HDACs by a retroviral protein was unknown. We recently showed that Vpr targets class I HDACs for proteasomal degradation in an inducible HeLa cell line. This function of Vpr was VprBP-dependent and correlated with the ability of Vpr to overcome latent infection of macrophages^28^. Here we show that Vpr also induces proteasomal degradation of class I HDACs in J-Lat cells, and primary T cells and this effect relied on the expression of VprBP. Similarly to our previous findings, HDAC3 was the most affected member of class I HDACs, followed by HDAC1 as the second most affected HDAC. HDAC2 and HDAC8 were only noticeably affected at MOIs as high as 5.0.

Counteraction of latency by the HIV-1 regulatory protein, Tat, is well documented. In fact, Tat is a viral transactivator that interacts with cellular proteins to relieve repression of the HIV-1 LTR^7^,^40^,^49^. On the other hand, Vpr is an accessory protein which is well known for its interaction with the E3-ubiquitin ligase complex and induction of proteasomal degradation of certain cellular proteins^23^,^25^,^50^. Several studies have also reported the ability of Vpr for transactivation of the HIV-1 LTR^51^,^52^,^53^,^54^. It was also reported that extracellular recombinant Vpr or serum Vpr isolated from HIV-1 positive patients are able to reactivate the latently infected primary cells and cell lines^34^,^55^. However, the detailed mechanism of Vpr-induced transactivation of LTR and involvement of the E3-ubiquitin ligase complex remained to be elucidated.

We showed that treatment of latently infected cells with Vpr peptide, which is frequently found in the serum of HIV-1-infected individuals, reactivated the virus. Vpr-induced reactivation of the latent HIV-1 infections was not resulted from Vpr-induced G2 arrest since blocking the G2 arrest did not abolish viral reactivation by Vpr. However, Vpr-induced reactivation of latently infected cells was found VprBP and proteasome dependent. In contrast to a previous report that found inhibition of proteasomal degradation using MG132 reactivates the latent provirus^56^, we did not observe viral reactivation when J-Lat cells were treated with MG132. This could be cell line dependent and indicate different mechanisms involved in establishment of latency in different cell lines.

Both HDAC1 and HDAC3 have been found to interact with HIV-1 LTR and their occupancy on the viral promoter is associated with HIV-1 latency^39^,^57^,^58^,^59^. Binding of HDAC1 to the latent HIV-1 LTR induces histone deacetylation and repressive changes in chromatin structure of the HIV-1 LTR^59^. Using different models for HIV-1 latency, recent studies suggest that HDAC3 plays a more important role in HIV-1 latency since depletion of HDAC3 results in a more significant viral reactivation^30^,^39^. In our study, we depleted HDAC1 and HDAC3, as the most affected HDACs by Vpr. Depletion of both proteins in two clones of J-Lat cells (10.6 and 6.3) reactivated the latent virus. Similarly to the previous studies, depletion of HDAC3 showed more potency in viral reactivation in our models, however this effect could be dependent on the cell-lines or the extent of depletion. We then focused on HDAC3 to further study its role in viral reactivation. Co-transduction of HDAC3 with Vpr, abrogated the ability of Vpr in viral reactivation (Fig. 4C), suggesting depletion of HDAC3 plays an important role in Vpr-induced viral reactivation. Interestingly, complementation with a mutant form of HDAC3, which was defective for deacetylase activity, did not compromise the ability of Vpr in viral reactivation, suggesting that the deacetylase activity of HDAC3 is essential for maintaining latency in HIV-1 provirus.

Previous studies have described transactivation of the HIV-1 LTR by Vpr^51^,^54^. Vpr-mediated transactivation of LTR was found independent of its cell cycle arrest activity. Ability of Vpr to increase viral replication was also directly correlated with the ability of Vpr to transactivate the HIV-1 LTR^51^. In order to directly address the role of Vpr in viral reactivation, we examined binding of Vpr to the HIV-1 LTR. We found that Vpr binds the HIV-1 LTR in a VprBP-dependent manner (Fig. 5), suggesting that Vpr-induced depletion of HDACs may be more specifically targeted towards the HIV-1 LTR. Interestingly, further experiments demonstrated that the wild-type Vpr, but not the Q65R mutant, induced depletion of HDAC1 and HDAC3 on the HIV-1 LTR (Fig. 6A), suggesting the VprBP dependency of this function of Vpr. Depletion of HDACs was accompanied by enrichment of acetylated histones and p65 on the HIV-1 LTR (Fig. 6B,C) which are markers of active HIV-1 transcription. Vpr-induced reactivation of the latent HIV-1 provirus was also linked to the enhanced recruitment of the cellular RNA polymerase II to the viral genome (Fig. 6D). It should also be noted that p65 is deacetylated by HDAC3^60^. Similarly, many other non-histone proteins are also targets for deacetylation by members of class I HDACs^61^,^62^,^63^ and therefore depletion of HDACs by Vpr may consequently affect the activity of those proteins.

In addition to the Vpr of the lab adapted strain, pNL4-3, which was used throughout the study, we compared the Vpr proteins of other strains for their ability to reactivate the latent HIV-1 provirus. We found that Vpr protein of other subtype B strains, as well as subtype A and C, were able to deplete HDAC1 and HDAC3 and reactivate the latent virus in J-Lat cells (Fig. 7). However, subtype A Vpr showed a lower expression and therefore only induced depletion of HDAC3, but not HDAC1. Interestingly, subtype A Vpr was still able to reactivate the latent provirus, suggesting that depletion of HDAC3 plays the main role in viral reactivation. This is consistent with previous studies indicating the predominant role of HDAC3 in viral silencing^30^,^39^.

It has been shown that the transactivating properties of Vpr is conserved among primate lentiviruses, while Vpx does not show a similar transactivating effect^54^. In addition, Vpr proteins of HIV-1 infected individuals have been previously reported to reactivate the latent virus in cell lines models for latency as well as latently infected primary cells^34^. In our study we found that Vpr isolated from patients also reactivated the latent provirus in J-Lat and U1 cells (Fig. 8A,B). Similarly, Vpr from patients reactivated the latent virus in PBMCs isolated from HIV-1-infected individuals with no detectable viral loads.

Collectively, our study shows that Vpr induces proteasomal degradation of class I HDACs, with HDAC3 as the major target for degradation. Vpr induced degradation of HDAC1 and HDAC3 in a VprBP-dependent manner which was linked to reactivation of latent HIV-1 provirus. Since Vpr is found in high quantities in the serum of HIV-1-infected patients, Vpr-induced degradation of class I HDACs, especially HDAC3, represents a bona fide mechanism for reactivation of latent HIV-1 reservoirs.

HDAC inhibitors currently serve as a promising strategy, known as shock and kill, to reactivate and purge the HIV-1 reservoir^20^,^29^. Our study suggests that the virus has already evolved a mechanism to reactivate latently infected cells by counteracting class I HDACs. Synergistic reactivation of HIV-1 expression by HDAC inhibitors and prostratin has been previously reported^64^. Since Vpr also reactivates the latent viruses by affecting HDACs, similar effects should be considered when viral reactivation is studied. Although expression of Vpr in J-Lat cells treated with SAHA enhanced viral reactivation similarly to SAHA treatment alone (Supplementary Fig. S2A), a synergy or interference between Vpr-induced viral reactivation and shock and kill strategy needs to be addressed in more details.

## Materials and Methods

### Cell lines and primary cells

HEK293T cells were obtained from ATCC. J-Lat full length cells (clone 10.6 and 6.3) and U1 cells were obtained from the NIH AIDS Research and Reference Reagent Program. J-Lat 10.6 was the only cell line when we refer to J-Lat cells unless otherwise stated J-Lat 6.3. All experiments were conducted in accordance with our internal ethics committee and were approved by the ethics committee at the Ahvaz Jundishapur University of Medical Sciences. Written informed consent was obtained from all subjects. PBMCs were isolated using Ficoll-density gradient centrifugation from HAART-treated HIV-1-infected individuals with no detectable viral loads and healthy donors. Primary CD4^+^ T cells were isolated from whole blood of healthy donors using Dynabeads CD4 (Thermo Fisher Scientific) according to the manufacturer’s instructions. CD4+ T cells were activated using 5 μg/mL PHA and 20 U/mL IL-2 for 3 days. Cell lines and primary cells were maintained in RPMI supplemented with 100 units/ml penicillin, 100 μg/ml streptomycin, and 10% FBS.

### Antibodies and reagents

HDAC1 (ab7028), HDAC2 (ab7029), HDAC3 (ab32369), HDAC8 (ab18968), histone H3 (ab70550), and HIV-1 Tat (ab43014) antibodies were purchased from Abcam. VprBP (A301-888A) antibody was from Bethyl Laboratories. Flag antibody (F3165) was from Sigma-Aldrich. GAPDH (14C10) and rabbit IgG isotype control (2729) were from Cell Signaling Technology. Anti-p24-PE (sc-69728 PE) was from Santa Cruz. Ace H3K9 (39917) and Ace H4K5 (39699) antibodies were from active motif. Mouse and rabbit HRP-conjugated antibodies were from Abcam. Monoclonal antibody against a conserved region of HIV-1 Vpr was raised in mouse by conjugating KLH (keyhole limpet hemocyanin) to a synthetic peptide (EAVRHFPRHWLHGLGQ) corresponding to amino acids 29–44 of Vpr prevalent among clinical isolates. SYBR Select Master Mix was from Life Technologies. Benzonase nuclease was from Novagen. Caffeine, Flag peptides, SAHA, and DMSO were from Sigma-Aldrich. MG132 was from Millipore. VprBP and non-targeting siRNAs were from Dharmacon. HDAC1 siRNA was from Qiagen. HDAC3 siRNA was from Santa Cruz Biotechnology. HDAC-Glo™ I/II Reagent was from Promega.

### Vectors and virus constructs

The lentiviral vectors, pWPI-Flag-Vpr, pWPI-Flag-Q65R, and pWPI-Flag-R80A, were described previously^35^. The WT and ∆Vpr HIV-1 vectors, namely pNL4.3.AD8. IRES_GFP_Nef- and pNL4.3.AD8. IRES_GFP_Nef-_Vpr-, were described previously. The lentiviral vector LeGO-iC2 that expresses IRES-mCherry was obtained from Addgene. Flag-Vpr and the corresponding mutants, Q65R and R80A, were amplified from pWPI-Flag-Vpr, pWPI-Flag-Q65R, and pWPI-Flag-R80A using Flag-Vpr-F 5′-AAGGATCCATGGATTACAAGGATGACGACGATAAG-3′ and Flag-Vpr-R 5′-TTGAATTCCTAGGATCTACTGGCTCCA-3′. The amplified fragments were then cloned into LeGO-iC2 using *Bam*HI and *Eco*RI restriction enzymes. pCMV-VSV-G, psPax2, mOrange-N1, pcDNA3.1-HDAC1-Flag, and pcDNA3.1-HDAC3-Flag were obtained from Addgene. To construct LeGO-iC2-IRES-mOrange, the IRES-mCherry from LeGO-iC2 was replaced with IRES-mOrange from mOrange-N1 using *Not*I and *Bsr*GI. HDAC1-Flag and HDAC3-Flag were amplified from pcDNA3.1-HDAC1-Flag and pcDNA3.1-HDAC3-Flag, respectively, using HDAC1-F 5′-AAGGATCCATGGCGCAGACGCAGGGCAC-3′, HDAC3-F 5′-AAGGATCCATGGCCAAGACCGTGGCCTA-3′ and HDAC1/3-R 5′-TTGAATTCTTACTTGTCATCGTCGTCCT-3′. The amplified fragments were cloned into LeGO-iC2-IRES-mOrange using *Bam*HI and *Eco*RI. Subtype B founder virus pWITO.c/2474, p89.6, subtype A clone p92UG037.1, and subtype C clone p93IN904 were obtained from the NIH AIDS Research and Reference Reagent Program. Vpr alleles were amplified and fused to Flag-tag from the full length HIV-1 clones using Vpr-subtype A/B-Forward 5′-AAGGATCCATGGATTACAAGGATGACGACGATAAGGAACAAGCCCCAGAAGA-3′.

Vpr-subtype C-Forward 5′-AAGGATCCATGGATTACAAGGATGACGACGATAAGGAACAACCCCCAGAAGA-3′.

Vpr-subtype B/C-Reverse 5′-TTGAATTCCTAGGATCTACTGGCTCCAT-3′ and Vpr-subtype A-Reverse 5′-TTGAATTCCTAGGATCTACCGGATCCAT-3′. Flag-tagged Vpr alleles were then cloned into LeGO-iC2 using *Bam*HI and *Eco*RI.

### Virus and lentiviral vectors production

HIV-1 viral particles and the lentiviral vectors were produced in HEK293T cells using the standard calcium-phosphate transfection method. Viral particles and lentiviral vectors were purified 48 h and 72 h post-transfection by ultracentrifugation at 35000 rpm for 2 h. Briefly, lentiviral vectors were produced by cotransfection of 40 μg of the LeGO-iC2-derived vectors, 6 μg pCMV-VSV-G and 15 μg psPax2. Lentiviral vectors were titrated in HEK293T cells using mCherry and mOrange signals. p24 of the concentrated viral stocks was titrated using ELISA.

### Vpr purification

To purify Flag-Vpr and Flag-Q65R, HEK293T cells were transduced with lentiviral vectors for expression of Flag-Vpr and Flag-Q65R. Cells were then lysed after 48 h and the Flag-tagged proteins were purified by affinity chromatography purification. To purify Vpr from patient’s serum, mouse antibody against Vpr was conjugated to chromatography columns and used for purification of Vpr from serum of HIV-positive individuals. Quality of the purified proteins was examined using 12% SDS-PAGE and Coomassie Blue staining.

### Viral lysis

HIV-1 viral particles were lysed in 0.5% Triton lysis buffer. Viral lysates were treated with 20 μg/ml RNase A to degrade viral RNA, preventing detection of the original viral genome by qPCR. Triton was then removed using Detergent Removal Spin Columns (Thermo Fisher Scientific). Viral lysates were tested using Western blot to ensure that the HIV-1 Tat protein is absent in the lysates.

### Treatment with extracellular Vpr

Five million J-Lat and U1 cells were treated with 10 ng/ml Flag-Vpr and Flag-Q65R or otherwise as stated. For the ChIP experiments, we treated J-Lat cells with 500 ng/ml Flag-Vpr or Flag-Q65R. In the time-course study, the treatment was repeated after 48 h and 100 μl of the supernatant was spared daily for the total of 6 days post-treatment. The amount of p24 was measured in the supernatant using ELISA. In order to study viral reactivation using viral lysates, 10^7^ unstimulated PBMCs isolated from HIV-1 infected individuals on HAART, as well as J-Lat cells, were treated with the WT and ∆Vpr viral lysates at the final concentration of 500 ng/ml p24. In the case of unstimulated PBMCs, the treatment was repeated after 48 h. To study the effect of clinical Vpr proteins isolated from the patients, Vpr was purified from the patients’ serum. Purified Vpr was added to the cells at the ratio of 1:100 such that it retained the original volume of the serum from which Vpr had been extracted.

### Lentiviral transduction

J-Lat cells (clone 10.6 and 6.3) were transduced with VSV-G pseudotyped lentiviral vectors using spinoculation method. To analyze depletion of HDACs, 10^7^ cells were transduced with the lentiviral vectors in 12-well plates. To analyze viral reactivation, 10^6^ cells were transduced with the lentiviral vectors in 24-well plates. Polybrene was added to the cells at the final concentration of 8 μg/ml and plates were centrifuged at 1500 g for 1 h at 25 °C. Cells were then transferred to 75 cm flasks or 6-well plates to study depletion of HDACs or viral reactivation, respectively.

### siRNA transfection

Specific siRNAs against HDAC1, HDAC3, and VprBP, as well as non-targeting siRNAs, were transfected in J-Lat cells using lipofectamine RNAiMAX Reagent according to the manufacturer instructions.

### Cell fractionation

J-Lat cells were transduced with lentiviral vectors and after 48 h, mCherry positive cells were sorted and lysed in 0.5% Triton lysis buffer (50 mM Tris, pH 7.5, 150 mM NaCl, 0.5% Triton, and protease inhibitor cocktail). Cells were incubated at 4 °C with mild agitation for 10 min. Cells were then centrifuged at 6000 RPM for 10 min at 4 °C to pellet chromatin and other large insoluble debris. Supernatant was transferred to a new tube as soluble fraction. The pellet was resuspended in 1 ml of Benzonase nuclease buffer (50 mM Tris pH 8, 150 mM NaCl, 1.5 mM MgCl_2_, 0.1 mg/ml BSA, and protease inhibitor cocktail) by pipetting followed by centrifugation at 6000 RPM for 10 min. The pellet was resuspended in 500 μl Benzonase nuclease buffer by pipetting and Benzonase was added to the final concentration of 25 U/ml. Samples were then incubated on ice for 1 h. Both the Benzonase-treated pellet and the soluble fraction were centrifuged at 13000 RPM for 10 min at 4 °C. Supernatants were then transferred to new tubes.

### Western blotting

Thirty μg of each cellular fraction or 3 × 10^5^ cells were resuspended in Laemmli buffer and heat-denatured for 5 min. Protein lysates were then run on 12% SDS-PAGE gels and Western blots were performed as described previously^35^.

### ELISA

In order to examine viral reactivation, supernatants of the cells were collected and inactivated with Triton X-100 (1% final concentration). HIV-1 p24 was quantified using HIV- 1 p24 Antigen Capture assay (ABL Inc.) according to the manufacturer’s instructions.

### Chromatin immunoprecipitation

To analyze enrichment of HDAC1, HDAC3, Vpr, p65, S2 phosphorylated RNAPII as well as acetylated histones on the HIV-1 LTR, J-Lat cells were treated with affinity purified Flag-Vpr and Flag-Q5R. GFP-positive cells were then sorted and fixed with 0.5% formaldehyde for 5 min. Fixation was quenched by adding glycine to a final concentration of 125 mM for 5 minutes. Cells were lysed and subjected to chromatin immunoprecipitation as previously described^28^.

### Quantitative PCR

Enrichment of Vpr, HDAC1, HDAC3, p65, S2 phosphorylated RNAPII, Ace H3K9 and Ace H4K5 on nuc-0 was analyzed using quantitative PCR. Enrichment of S2 phosphorylated RNAPII was additionally analysed on regions covering nucleotide 2000, 4000, 6000, and 8000 of the HIV-1 genome. Briefly, the chromatin immunoprecipitated DNA and the corresponding input DNA were subjected to PCR using the following primers: GAPDH-F 5′-CCTCCTTCCCCTAGTCCCCAGAA-3′, GAPDH-R 5′-GAGCGCGAAAGGAAAGAAAGCGT-3′, nuc-0-F 5′-CCACACACAAGGCTACTTCCCT-3′, nuc-0-R 5′-CAACTGGTACTAGCTTGTAGCA-3′, nuc-1-F 5′-GTCTCTCTGGTTAGACCAGA-3′, nuc-1-R 5′- TACTTGAAGCACTCAAGGCA -3′,

HIV-2000-F 5′-ATTGTTAAGTGTTTCAATTGTGGCA-3′,

HIV-2000-R 5′-CTTGTAGGAAGGCCAGATCTTCCCT-3′,

HIV-4000-F 5′-CTAACTGACACAACAAATCAGAAGA-3′,

HIV-4000-R 5′-TTTGATTGACTAACTCTGATTCACT-3′,

HIV-6000-F 5′-GCCTTAGGCATCTCCTATGGCAGGA-3′,

HIV-6000-R 5′-TGCTACTACTAATGCTACTATTGCT-3′,

HIV-8000-F 5′-GGCAAGAATCCTGGCTGTGGAAAGA-3′,

HIV-8000-R 5′-ATCCAGGTCGTGTGATTCCAAATCT-3′. An intergenic region, where no hyperacetylation of histones is expected (gene accession number: AF254641.1), was amplified using INT-F 5′-GTAGAGGAAGCGATCTGGGA-3′, INT-R 5′-CAAGGCCACTCTCGGCCTCT-3′. Amplification of the target sequences was detected using SYBR Select Master Mix, and normalized to the input. Enrichments were calculated based on the fold enrichments relative to the intergenic region. In order to detect HIV-1 RNA in the supernatant, viral RNA was extracted using Ultrasens Viral RNA kit (Qiagen) and reverse transcribed using SuperScript II Reverse Transcriptase and oligo(dT)12–18 primers. By aligning the available sequences of the local HIV-1 strains (Los Alamos database) with pNL4-3, HIV-1-env-F 5′-GGACAAGCAATGTATGCTCCTCCCA-3′ and HIV-1-env-R 5′-GGTGGGTGCTACTCCTAGTG GTTCA-3′ primers were designed. pNL4-3 was used to quantitate viral copies and along with cDNA was subjected to quantitative PCR using HIV-1-env-F and HIV-1-env-R primers.

### Flow cytometry and sorting

J-Lat cells were transduced with VSV-G pseudotyped lentiviral vectors expressing wild-type Vpr, or the Q65R and R80A mutants. The GFP signal of the activated provirus was analyzed 24 h post-transduction using CyAn ADP Analyzer. In order to analyze expression of viral capsid in activated cells, 48 h-post transduction cells were permeabilized and intracellular p24 was labeled using Anti-p24-PE. To measure viral activation by soluble Vpr, J-Lat cells were treated with affinity purified Vpr and expression of GFP was analyzed using flow cytometry. To analyze the cell cycle profile of J-Lat cells, they were transduced with VSV-G pseudotyped lentiviral vectors expressing Vpr. After 48 h, the DNA content of the cells was labeled by propidium iodide staining and cells were analyzed as previously described^35^. Transduced J-Lat cells expressing mCherry were sorted using an Influx cell sorter (BD Biosciences) and analyzed by Western blot. GFP-positive J-Lat cells expressing the activated provirus were also sorted and analyzed using ChIP assay.

### Deacetylase activity

HEK293T cells were transduced with VSV-G psuodotyped lentiviral vectors expressing the Flag-tagged wild-type or the H134/135A mutant HDAC3. After 48 h, cells were lysed and Flag-tagged proteins were purified using anti-Flag-agarose beads. The histone deacetylase activities of the wild-type and H134/135A HDAC3 were measured using HDAC-Glo™ I/II Reagent as described by the manufacturer.

### Statistical Analysis

Student’s *t* test was used for the statistical analysis when 2 sets of data were compared. Analysis of variance (ANOVA) was used for comparison of more than 2 sets of data. The statistical analyses were performed using GraphPad Prism 6.0. A value of *p* < 0.05 was considered statistically significant. Results were expressed as mean S.D., and represent data from a minimum of three independent experiments unless otherwise stated.

## Additional Information

**How to cite this article**: Romani, B. *et al*. HIV-1 Vpr reactivates latent HIV-1 provirus by inducing depletion of class I HDACs on chromatin. *Sci. Rep.* **6**, 31924; doi: 10.1038/srep31924 (2016).

## Supplementary Material

Supplementary Information
