# Supplementary material for: HIV-1 Vpr reactivates latent HIV-1 provirus by inducing depletion of class I HDACs on chromatin
Source: Sci Rep. 2016 Aug 23;6:31924. doi: 10.1038/srep31924 (PMC4994036; doi:10.1038/srep31924)
Supplement: Supplementary Information [file srep31924-s1.pdf]

HIV-1 Vpr reactivates latent HIV-1 provirus by inducing depletion of class I HDACs on chromatin

Bizhan Romani[1,2], Razieh Kamali Jamil[3], Mojtaba Hamidi-Fard[4], Pooneh Rahimi[4], Seyed Bahman Momen[5], Mohammad Reza Aghasadeghi[4*], and Elham Allahbakhshi[1]**

[1]Cellular and Molecular Research Center (CMRC), Faculty of Medicine, Ahvaz Jundishapur University of Medical Sciences (AJUMS), Ahvaz, 61357-15794, Iran

[2]Department of Biology, Faculty of Science, University of Isfahan, Isfahan, 81746-73441, Iran

[3]Department of Human Viral Vaccines, Razi Vaccine and Serum Research Institute, Karaj, 31976-19751, Iran

[4]Hepatitis and AIDS Department, Pasteur Institute of Iran, Tehran, 13169-43551, Iran

[5]Pilot Biotechnology Department, Pasteur Institute of Iran, Tehran, 13169-43551, Iran

***Corresponding author:** Mohammad Reza Aghasadeghi

Tel.: +98 216 696 9291; Fax: +98 216 696 9291; E-mail: mrasadeghi@pasteur.ac.ir

****Corresponding author:** Elham Allahbakhshi

Tel: +98 916 113 7679; Fax: +98 613 333 6380; E-mail: abakhshi_e@ajums.ac.ir

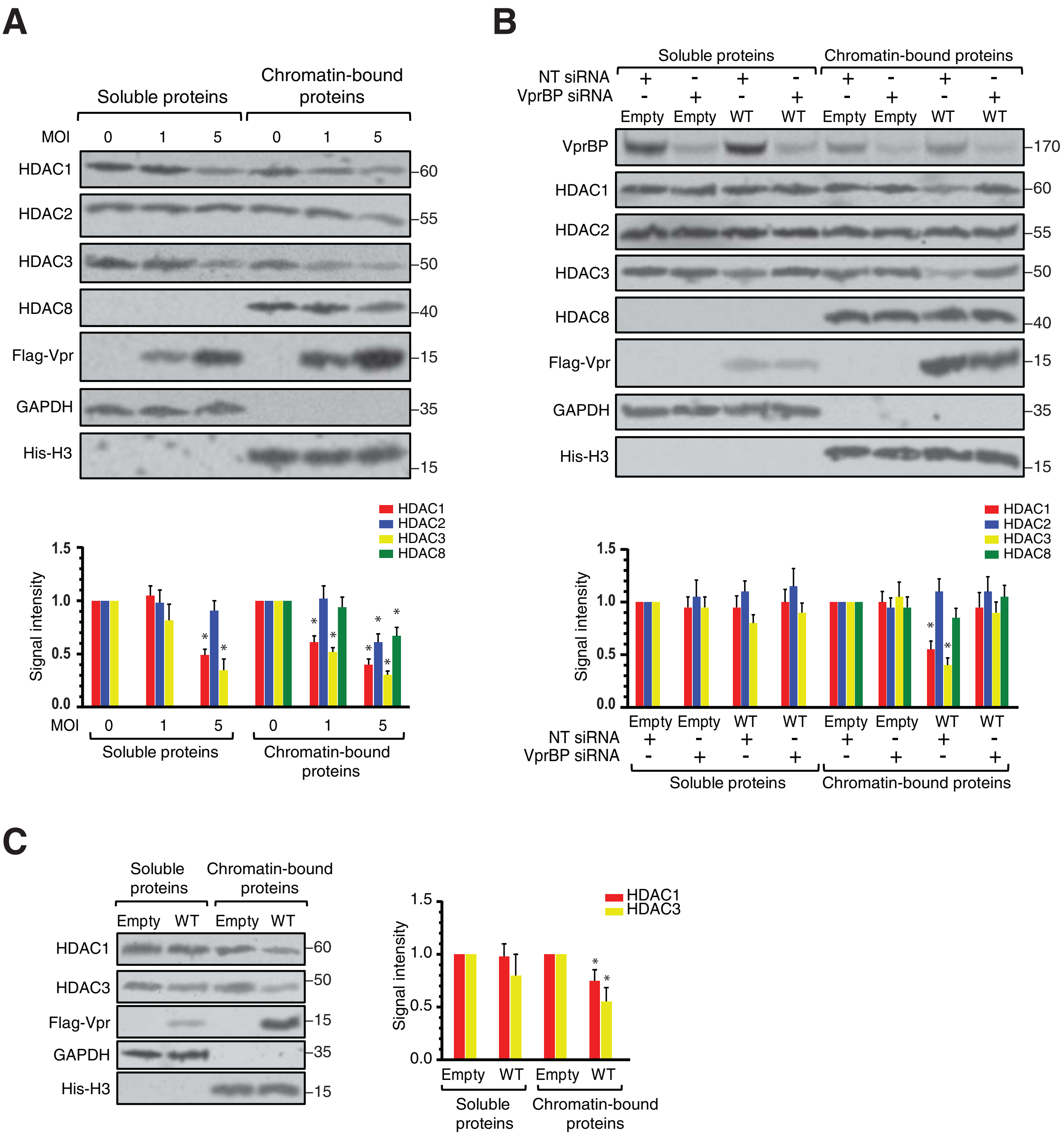
A
Soluble proteins
Chromatin-bound proteins
MOI 0 1 5 0 1 5
HDAC1 -60
HDAC2 -55
HDAC3 -50
HDAC8 -40
Flag-Vpr -15
GAPDH -35
His-H3 -15
HDAC1
HDAC2
HDAC3
HDAC8
Signal intensity
1.5
1.0
0.5
0.0
MOI 0 1 5 0 1 5
Soluble proteins
Chromatin-bound proteins
B
Soluble proteins
Chromatin-bound proteins
NT siRNA + - + - + - + -
VprBP siRNA - + - + - + - +
Empty Empty WT WT Empty Empty WT WT
VprBP -170
HDAC1 -60
HDAC2 -55
HDAC3 -50
HDAC8 -40
Flag-Vpr -15
GAPDH -35
His-H3 -15
HDAC1
HDAC2
HDAC3
HDAC8
Signal intensity
1.5
1.0
0.5
0.0
Empty Empty WT WT Empty Empty WT WT
NT siRNA + - + - + - + -
VprBP siRNA - + - + - + - +
Soluble proteins
Chromatin-bound proteins
C
Soluble proteins
Chromatin-bound proteins
Empty WT Empty WT
HDAC1 -60
HDAC3 -50
Flag-Vpr -15
GAPDH -35
His-H3 -15
HDAC1
HDAC3
Signal intensity
1.5
1.0
0.5
0.0
Empty WT Empty WT
Soluble proteins
Chromatin-bound proteins

Figure S1. Depletion of class I HDACs by Vpr.

(A) J-Lat cells were transduced at an MOI of 1.0 and 5.0 with VSV-G pseudotyped mCherry-marked lentiviral vectors expressing Flag-Vpr. The total MOI was adjusted to 5.0 by adding empty lentiviral vector. After 48 h, mCherry expressing cells were sorted and fractionated.

(B) J-Lat cells were transfected with VprBP or non-targeting siRNAs. After 24 h cells were transduced with VSV-G pseudotyped mCherry-marked lentiviral vectors for expression of Flag-Vpr. Transduced cells were sorted and fractionated 48 h post-transduction.

(C) PHA+IL-2-activated $CD4^+$ T cells were transduced at an MOI of 1.0 with mCherry-marked lentiviral vectors expressing Vpr. Transduced cells were sorted 48 h post-transduction and fractionated.

All experiments were repeated 3 times.

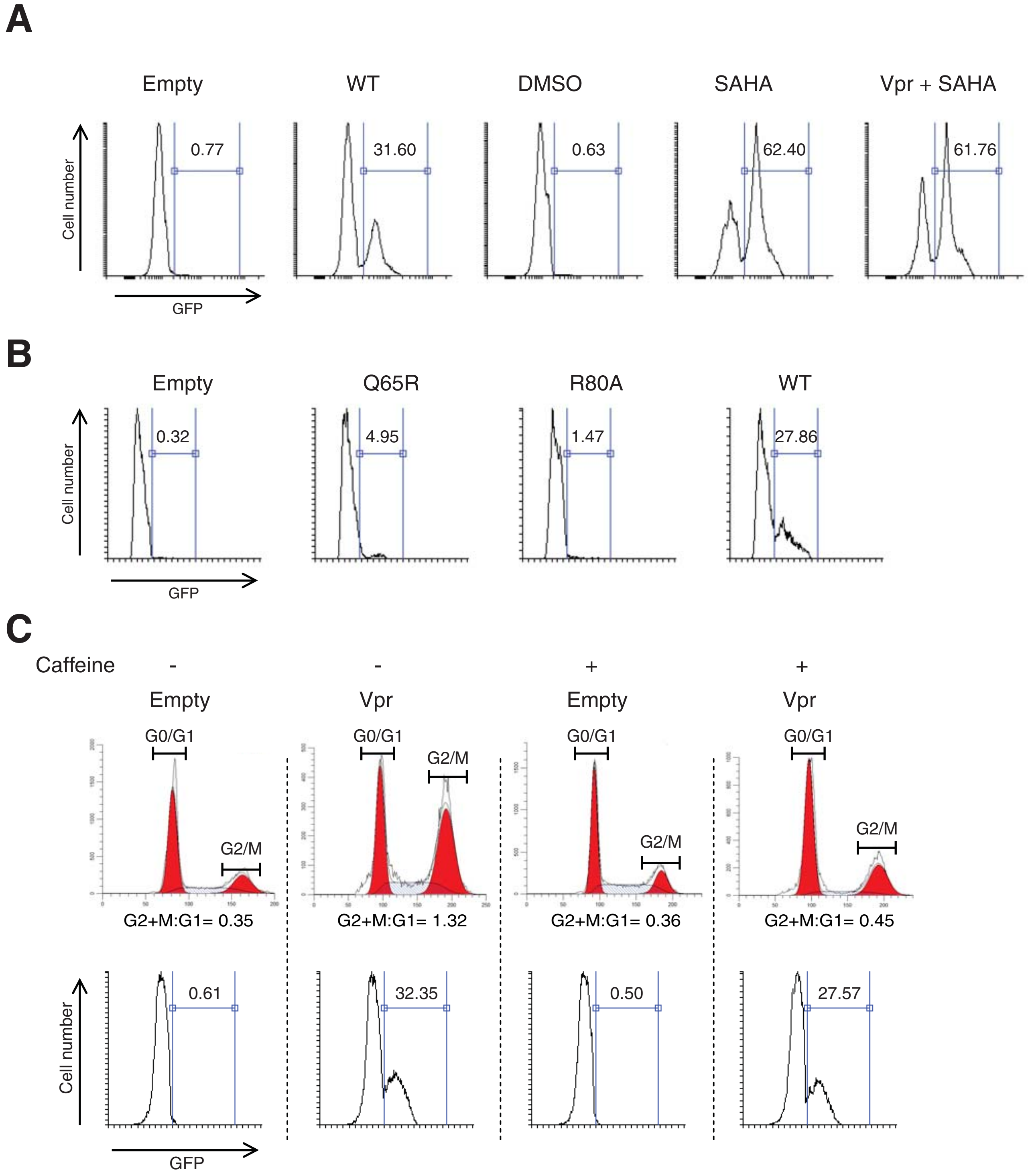
A
Empty
WT
DMSO
SAHA
Vpr + SAHA
0.77
31.60
0.63
62.40
61.76
Cell number
GFP
B
Empty
Q65R
R80A
WT
0.32
4.95
1.47
27.86
Cell number
GFP
C
Caffeine
-
-
+
+
Empty
Vpr
Empty
Vpr
G0/G1
G2/M
G2+M:G1= 0.35
G2+M:G1= 1.32
G2+M:G1= 0.36
G2+M:G1= 0.45
0.61
32.35
0.50
27.57
Cell number
GFP

Figure S2. Vpr-induced reactivation of HIV-1 latent provirus is not dependent on cell line or G2 cell cycle arrest.

(A) J-Lat 10.6 cells were transduced with empty or Vpr-expressing lentiviral vector or treated with SAHA and DMSO. J-Lat cells were also transduced with Vpr-expressing lentiviral vector and immediately treated with SAHA. Twenty four hours after transduction or treatment, cells were analyzed for viral reactivation using flow cytometry. The experiment was repeated 2 times.

(B) J-Lat 6.3 cells were transduced with lentiviral vectors for expression of the wild type or mutant Vpr proteins. After 24 h, cells were analyzed for expression GFP. The experiment was repeated 2 times.

(C) J-Lat 10.6 cells were transduced with lentiviral vectors for expression of Vpr or empty vectors. Cells were also treated with caffeine at the same time as transduction. After 24 h, the cell cycle profile (upper panels) and viral reactivation (lower panels) were analyzed. The experiment was repeated 2 times.

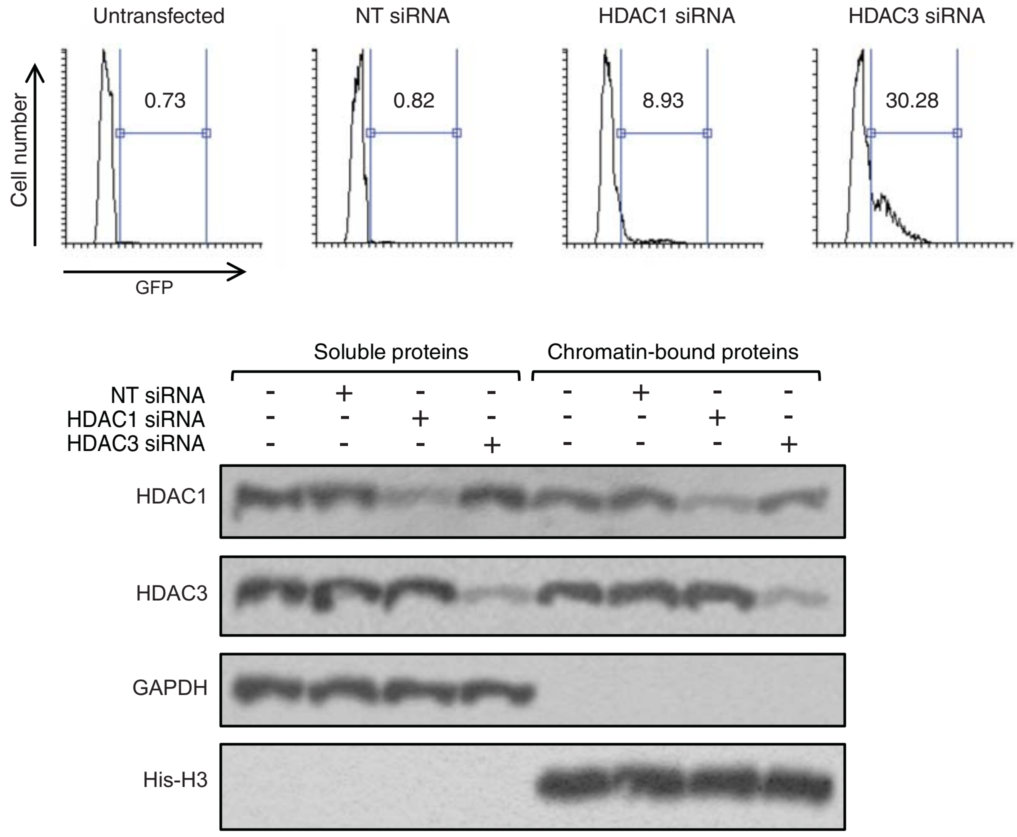

Figure S3. Depletion of HDAC1 and HDAC3 reactivates the latent provirus in J-Lat 6.3.

J-Lat 6.3 cells were transfected with siRNAs against HDAC1 and HDAC3. As control, J-Lat 6.3 cells were also transfected with non-targeting siRNAs (NT). After 48 h, cells were analyzed for expression of GFP (the upper panel). Some cells were also fractionated and analyzed using Western blot (the lower panel).

A

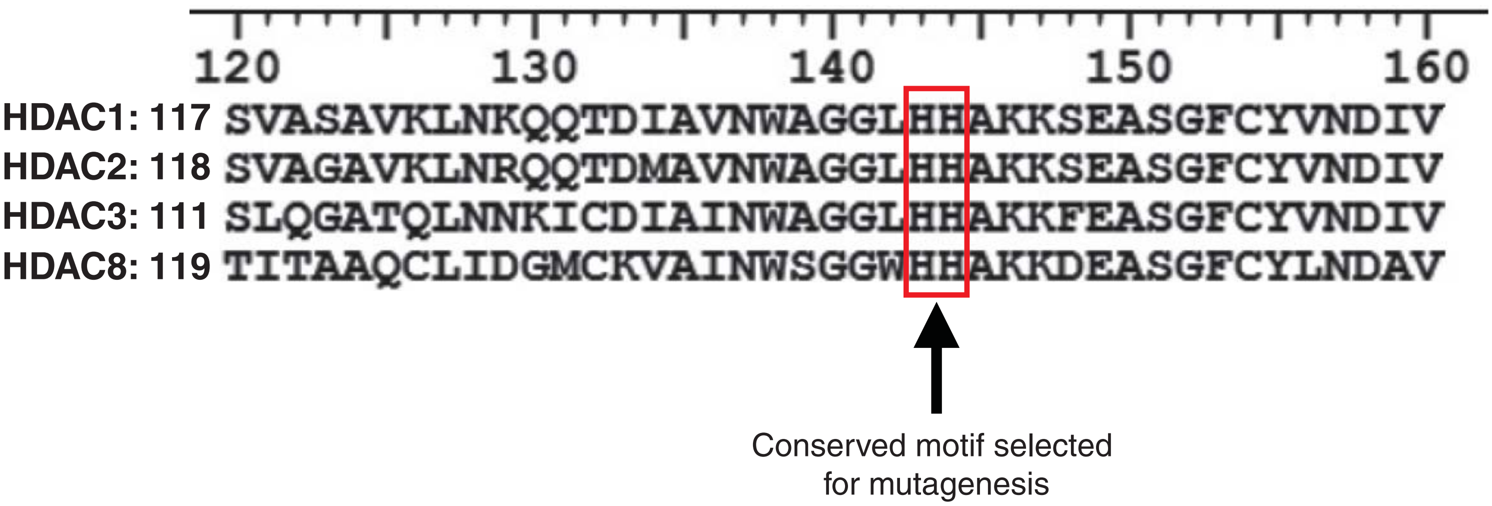

B

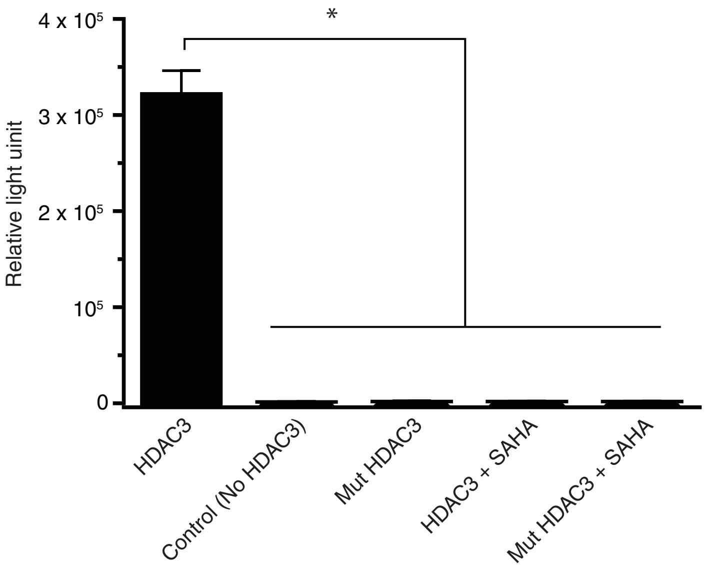

Figure S4. H134/135A HDAC3-Flag and its histone deacetylase activity.

(A) Alignment of class I HDACs showing a conserved motif selected for generating a mutant HDAC3

(B) Comparing histone deacetylase activity of HDAC3 and H134/135A HDAC3-Flag (mut-HDAC3). HEK293T cells were transfected with Flag-tagged HDAC3 or mut-HDAC3. After 48 h, cells were lysed and HDAC3 or mut-HDAC3 were affinity purified. Purified proteins were splitted in two aliquots and treated with SAHA or DMSO. Histone deacetylase activities of the purified HDAC3 and mut-HDAC3 were measured as relative light units. The experiment was repeated 3 times.

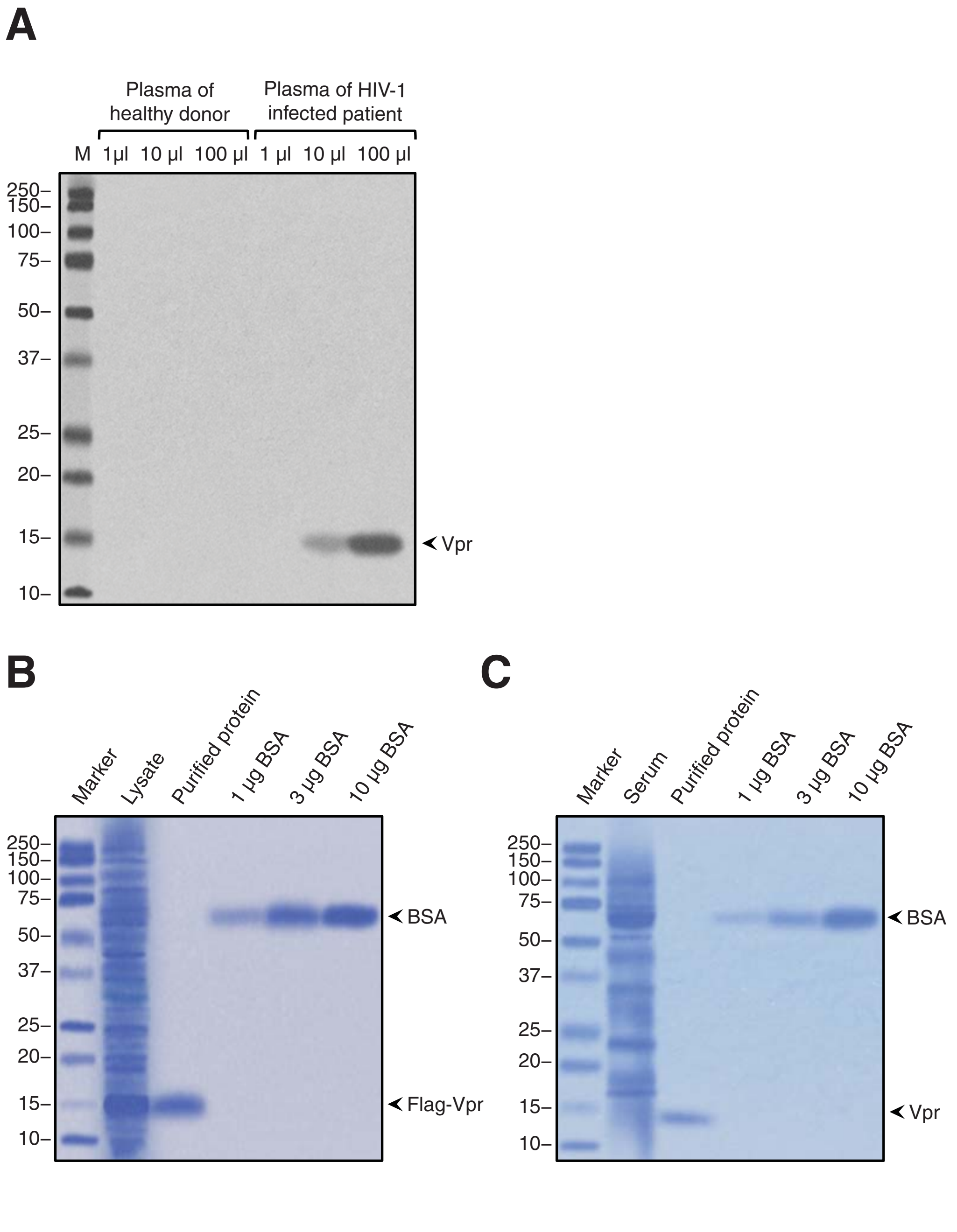

Figure S5. Characterization of Vpr antibody and efficiency of Vpr purification.

(A) Plasma was isolated from a healthy donor and an HIV-1 infected patient. Different amounts of plasma were analyzed by Western blot using the homemade antibody.

(B) HEK293T cells were transduced with lentiviral vectors expressing Flag-Vpr. After 48 h, Flag-Vpr was purified from the cell lysate by affinity chromatography purification and loaded on SDS-PAGE. Gel was stained using Coomassie Blue staining.

(C) Vpr from serum of HIV-positive individuals was purified using home-made Vpr antibody conjugated to chromatography columns. Serum-purified Vpr was loaded on SDS-PAGE and stained using Coomassie Blue staining.

(D) J-Lat 10.6 cells were treated with 500 ng/ml Flag-Vpr and serum purified Vpr. After 24 h, cells were analyzed using flow cytometry for expression of GFP.

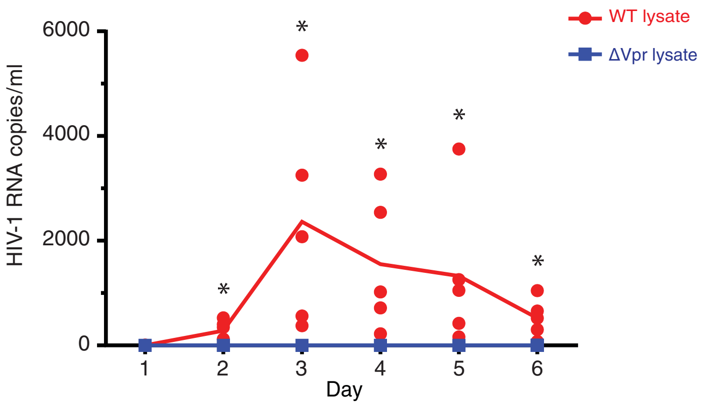

Figure S6. Release of virion-incorporated Vpr from HIV-1 virions reactivates latently infected cells.

PBMCs were isolated from five HAART-treated HIV-1 infected individuals with no detectable viral load. Unstimulated PBMCs were then treated with viral lysates of the WT or ΔVpr HIV-1. Viral load was measured in the supernatant by quantifying the HIV-1 RNA using quantitative PCR. Asterisks (*) indicate statistically significant differences between the WT and ΔVpr lysate treated cells.
